# Comparative *In Vivo* Analysis of Recombinant Type II Feline Coronaviruses with Truncated and Completed ORF3 Region

**DOI:** 10.1371/journal.pone.0088758

**Published:** 2014-02-20

**Authors:** Ádám Bálint, Attila Farsang, Zoltán Zádori, Sándor Belák

**Affiliations:** 1 National Food Chain Safety Office Veterinary Diagnostic Directorate, Budapest, Hungary; 2 National Food Chain Safety Office Directorate of Veterinary Medicinal Products, Budapest, Hungary; 3 Institute for Veterinary Medical Research, Centre for Agricultural Research, Hungarian Academy of Sciences, Budapest, Hungary; 4 Department of Virology, Immunobiology and Parasitology, National Veterinary Institute, SVA, Uppsala, Sweden; University of Hong Kong, China

## Abstract

Our previous *in vitro* comparative study on a feline coronavirus (FCoV) pair, differing only in the intactness of their ORF3abc regions, showed that the truncated ORF3abc plays an important role in the efficient macrophage/monocyte tropism of type II feline infectious peritonitis virus (FIPV). In the present study, we describe a challenge experiment with the same recombinant FCoVs in order to gain data on the *in vivo* characteristics on these viruses. While parent virus FIPV DF-2 developed feline infectious peritonitis in all the infected cats, its recombinant virus PBFIPV-DF-2, differing only in seven nucleotides, proved to be surprisingly low virulent, although caused an acute febrile episode similarly to the original FIPV DF-2. PBFIPV-DF-2 infection induced significantly lower virus neutralization titers than its parent virus, and lacked the second phase of viremia and development of fatal course of the disease. The recombinant PBFIPV-DF-2-R3i with completed ORF3abc gained biological properties that differentiate between the feline enteric coronavirus (FECV) and FIPV biotypes such as intensive replication in the gut, absence of viremia and weak or no serological response. Using reverse genetic approaches our study is the first experimental proof that ORF3abc is indeed responsible for the restriction of FECV replication to the intestine *in vivo*.

## Introduction

Feline coronaviruses (FCoVs), members of the *Alphacoronavirus* genus within the *Coronaviridae* family, are major pathogens of *Felidae* with worldwide distribution [1]. FCoV occurs in two pathotypes; feline enteric coronavirus (FECV) primarily replicates in the lower portion of intestinal tract, spreads by fecal-oral route, and its clinical appearance is characterized by mild or unapparent enteritis [2], [3]. In contrast, feline infectious peritonitis virus (FIPV) efficiently replicates in macrophages/monocytes, and can lead to feline infectious peritonitis (FIP), a highly lethal systemic granulomatous disease, [4]–[8].

FIPVs arise most likely from FECV in the infected cat via genetic changes [9]. Characteristic changes can be detected in the spike (S) gene [10], [11], in the ORF7ab [9], [12], [13] and the ORF3abc [9], [14]–[16] regions. FECVs have three open reading frames (ORFs) in the ORF3abc region [6] that code proteins conserved both in length and sequence in different isolates. On the contrary, the majority of FIPVs contain genetic alterations (non-synonymous mutations, deletions and termination codons) mostly in ORF3c but not rarely in ORF3a and ORF3b [9], [14].

The first *in vitro* comparison of a recombinant FCoV pair differing only in the intactness of their ORF3abc revealed that completion of the truncated ORF3abc reduces virus replication rate by 2log_10_ titer in feline peripheral blood monocytes [17] supporting the long time suspected but never experimentally proved theory that completion of this region alters the *in vivo* characteristics and pathogenesis of FCoV [8].

In the present study using the parent FIPV DF-2 strain and its recombinant derivates we aimed to collect *in vivo* data how the completed ORF3abc alters virulence, virus shedding, viremia, viral load of organs and humoral immune response against type II FCoV. The data of our experiments show that completion of ORF3abc vested the highly virulent FIPV DF-2 with properties that are characteristic to FECV.

## Materials and Methods

### Cells and Viruses

*Felis catus* whole fetus 4 (FCWF-4) cells originally purchased from the American Type Culture Collection were used for virus propagation, titration and virus neutralization tests. The cell line was maintained as monolayer culture in Dulbecco’s Modified Eagle Medium (Sigma-Aldrich, Saint Louis, MO, USA) supplemented with 10% fetal bovine serum (FBS), 0.3 mg/ml glutamine, 100 U/ml penicillin, 0.1 mg/ml streptomycin, 0.25 µg/ml amphotericin B, 1 mM sodium pyruvate, and 1% non-essential amino acids (Sigma-Aldrich). The FIPV DF-2 strain was kindly provided by Berndt Klingeborn (SVA, Uppsala, Sweden). FIPV DF-2 is a regular tissue culture adapted strain that has been well described in the literature, and also used by many other investigators under this name or as FIPV-79-1146 or FIPV-Nor15 [16]. Generation of the recombinant PBFIPV-DF-2 and PBFIPV-DF-2-R3i was described elsewhere [17]. Briefly, PBFIPV-DF-2 is a virus that originated as a molecular clone of FIPV DF-2 and then was successfully transfected into cat cells, where it was replicated for several generations before use in this study. PBFIPV-DF-2-R3i is a derivate of PBFIPV-DF-2 that was re-engineered to contain the intact ORF3abc region of canine coronavirus, and was also transfected into cat cells and cultivated for several generations before being used in this study.

### Sequence Analysis

The complete genome of PBFIPV-DF-2 was reverse transcribed using the high fidelity SuperScript III First-Strand Synthesis System (Invitrogen, Carlsbad, CA, USA) and gene-specific primers. Long PCR fragments overlapping the whole genome were amplified with Phusion Hot Start High-Fidelity DNA Polymerase (Finnzymes, Espoo, Finland) and sequenced using the Ion Proton System (Life Technologies, Carlsbad, CA, USA). Sequences were aligned and analyzed with the SeqMan Ngen software (Lasergene, Madison, WI, USA).

### Animal Experiments

Specific-pathogen-free IQHsdCpb kittens (Isoquimen SL, Barcelona, Spain) were used in the challenge experiments. Kittens arrived at the facility at the age of 8–12 weeks. They were acclimated and used in the studies at the age of 14–18 weeks. The animals were kept in separate groups in a closed facility. Their FCoV negative status was checked with PCR, ELISA and virus neutralization tests. Kittens were inoculated oronasally with 10^3^ 50% tissue culture infective doses (TCID_50_) of the parent virus FIPV DF-2 (n = 4) and the recombinant viruses PFIPV-DF-2 (n = 4) and PFIPV-FD-2-R3i (n = 4), respectively. Kittens were clinically examined on a daily basis for 42 days. Cats were scored for several clinical signs as described earlier [18]. Briefly, scoring was based on depression (inactivity for three consecutive days, 1 point), anorexia (not eating for three consecutive days, 1 point), and neurological disorders (swaggering, 1 point) on a daily basis, while fever (40.1°C, 1 point), jaundice (yellow plasma, 1 point), weight loss (loss of 2.5% of body weight per week, 1 point), and lymphopenia (lymphocyte count of <0.5×10^9^/liter) was scored on weekly basis. Kittens showing signs of terminal FIP were euthanized in order to avoid unnecessary suffering, while healthy animals were exterminated at day 42 postinfection (p.i.), followed by full postmortem examination. All animal experiments were approved and supervised by the Ethical and Animal Welfare Committee of National Food Chain Safety Office (Permission No: 2866/2011). The total number of animals was carefully determined by considering two main principles: i.) the number of animals should be ensured proper amount of samples for statistical analysis and ii.) the 3R rules (Replacement, Reduction and Refinement) must be implemented.

### Detection of Virus Shedding

To determine virus shedding in feces, fecal swabs were collected at days 0, 7, 14, 21, 28, 35 and 42 p.i., and placed in 500 µl of phosphate buffered saline (PBS). After vortexing and 30 min incubation, the swabs were removed, and the extract was centrifuged at 1000 x g for 10 min to remove cell debris. The supernatant was used for subsequent PCR.

Viral RNA was purified using the QIAamp Viral RNA Mini Kit (Qiagen, Hilden Germany). All RNA was stored at -80°C until used. To measure the copy numbers of the genome and replicative forms of CoVs, two TaqMan-based quantitative real-time PCR (qRT-PCR) assays targeting the 5′ end of the FIPV DF-2 genome and the N gene subgenomic (sg) mRNA were applied, respectively [17]. Each RNA sample was analyzed in duplicates in two different runs. Differences in original template RNA levels were normalized by using housekeeping gene β-actin PCR [19]. Means of the four normalized data per sample were used for further analysis.

### Detection of Viremia

RNA was extracted from whole EDTA anticoagulated blood taken at days 0, 7, 14, 21, 28, 35 and 42 p.i. using the QIAamp RNA Blood Mini Kit (Qiagen) according to the manufacturer’s instructions, and was subjected to genomic and subgenomic qRT-PCRs.

### Viral Load of Organs

To detect virus load in different organs (liver, spleen, kidney, lung, tonsil, mesenteric lymph nodes, brain and ileum,) approximately 0.5 g pieces of organs diluted in sterile phosphate-buffered saline (PBS) were homogenized with Tissue Lyser (Qiagen, Hilden, Germany) to obtain 50% w/v suspension and then were centrifuged at 1000 x g for 10 min to remove cell debris. RNA was extracted from the supernatant using the QIAamp Viral RNA Mini Kit (Qiagen), and was subjected to subsequent genomic and subgenomic qRT-PCRs.

### Serological Assays

Serum samples were taken using Vacuette® tube (Greiner Bio-One, Germany) at days 0, 7, 14, 21, 28, 35 and 42 p.i. For antibody ELISA tests, the FCoV EIA Kit, (BV European Veterinary Laboratory, The Netherlands) was used according to the recommendations of the manufacturer.

For virus neutralization (VN) assay, two-fold dilutions of heat-inactivated serum from kittens (50 µl) were incubated for 1 hour at 37°C with equal aliquots of FIPV DF-2 (50 µl of 10^3.5^ TCID_50_/ml). The viruses were then added to FCWF-4 cells showing 70% confluency in a 96-well plate, and incubated for 48 h, until the development of cytopathic effect. Neutralizing activity was determined by end-point dilution [20].

### Statistical Analysis

To determine the statistically significant difference between the VN titers generated after inoculation with the three FCoVs, the unpaired two-tailed Student T test with equal variances was applied. The p value under 0.05 was considered as a statistically significant difference.

## Results

### Virulence of Recombinant Viruses

Cats inoculated with the parent virus FIPV-DF-2 showed rapid development of FIP at day 10–16 p.i. The animals exhibited depression and anorexia, in most cases with fever, jaundice, weight loss and lymphopenia, and they had to be euthanized between days 21–25 (Table 1). Pathological examinations proved the characteristic lesions of effusive FIP with multiple dispersed pyogranulomas in the abdominal organs such as livers, spleens and kidneys.

**Table 1:** Total clinical scores of cats challenged oronasally with the parent virus FIPV DF-2 (n = 4) and recombinant FCoVs PBFIPV-DF-2 (n = 4) and PBFIPV-DF-2-R3i (n = 4).

Virus andanimal no.	Clinical score							Total clinical score	Day of death postinfection
	Fever	Depression	Anorexia	Jaundice	Neurological disorder	Weight loss	Lymphopenia		
FIPV DF-2									
1	2	2	2	3	1	3	2	15	21
2	2	2	2	3	0	2	2	13	25
3	2	3	3	3	1	3	2	17	21
4	2	2	2	3	1	2	2	14	22
PBFIPV- DF-2									
5	1	1	1	0	0	1	1	5	–
6	1	1	1	0	0	1	1	5	–
7	1	1	0	0	0	0	0	2	–
8	1	1	1	0	0	0	1	4	–
PBFIPV-DF-2-R3i									
9	0	0	0	0	0	0	0	0	–
10	0	0	0	0	0	0	0	0	–
11	0	0	0	0	0	0	0	0	–
12	0	0	0	0	0	0	0	0	–

Surprisingly, cats challenged with PBFIPV-DF-2, a recombinant FCoV containing truncated ORF3abc like the parent virus, showed only clinical signs of the acute phase of the disease, including transient fever from day 3 to 8, anorexia and slight lymphopenia (Table 1). All cats fully recovered and survived until termination of the experiment (day 42 p.i.). Macroscopically no lesions were observed in these animals.

Cats inoculated with PBFIPV-DF-2-R3i, the recombinant FCoV containing complemented ORF3abc, showed neither any clinical signs typical of FIP nor diarrhea (Table 1). All cats survived, and showed no macroscopic lesions except for slight enlargement of mesenteric lymph nodes in two animals.

In order to elucidate the unexpected low-virulent phenotype of PBFIPV-DF-2, the full-length genomic sequence of the virion was determined using next generation sequencing, and data revealed that besides the 1-nucleotid (nt) change at position 24429 (G/A) that resulted in an amino acid (aa) change in the fusion domain of S protein at position 1332 (V/I) and the 1-nt silent mutation at position 26064 (T/C) in the M gene found also in the infectious clone [17], additional mutations are present in the viral genome. In the ORF1ab gene, three nucleotide substitutions were found at positions 3098 (A/G), 5241 (G/A) and 7632 (C/T) resulting in aa changes at positions 930 (T/A), 1644 (G/D) and 2441 (S/L) affecting non-structural proteins (nsps) 3 and 4. Furthermore, a single nt change was present at position 27817 (C/G) affecting the last nucleotide of ORF 7 transcription regulatory sequence (TRS), and a 1-nt substitution also occurred at position 28492 (G/C) causing an amino acid change at position 121 (K/N) in the 7b protein. The possible role of mutation of the TRS of ORF7 in decreased ORF7 mRNA transcription was examined by an ORF7-specific subgenomic qRT-PCR assay, and similar subgenomic ORF7 mRNA levels were detected after inoculating FCWF cells with the wild type and recombinant FCoVs, indicating no effect of this mutation to mRNA transcription (data not shown). The genome of PBFIPV-DF-2-R3i contained only the point mutations observed in the infectious clone.

### Virus Shedding

**Figure 1:**
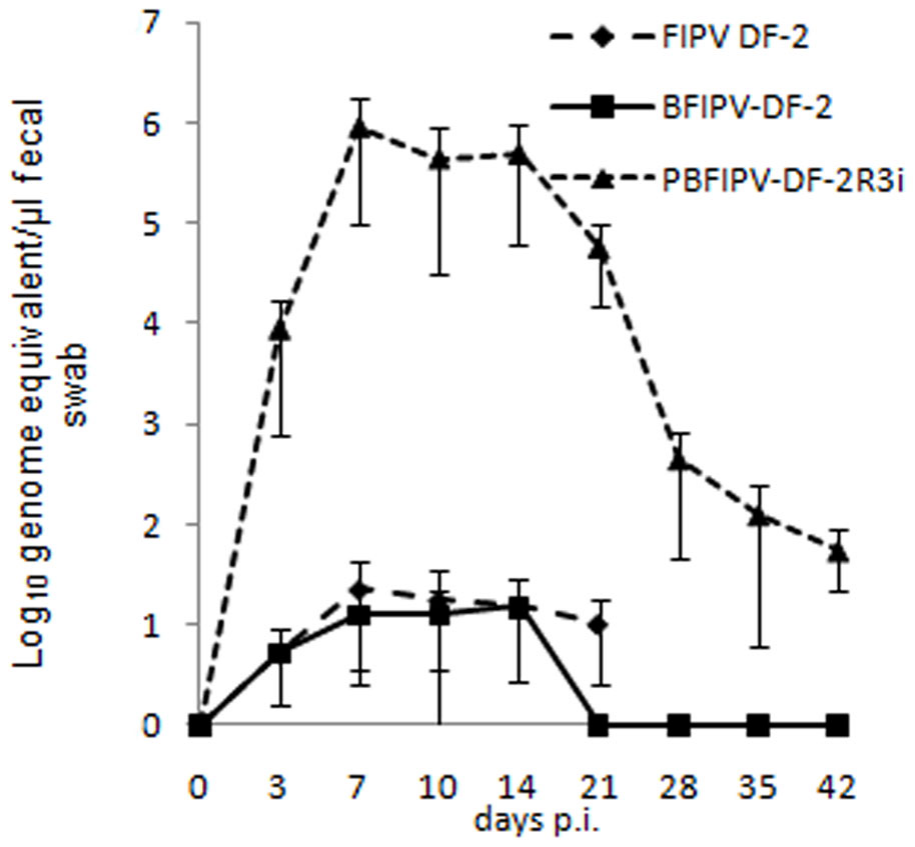
Fecal shedding of FCoV by cats challenged oronasally with the parent virus FIPV DF-2 and recombinant FCoVs PBFIPV-DF-2 (n = 4) and PBFIPV-DF-2-R3i (n = 4), as monitored with genomic qRT-PCR. The means of groups are given. Error bars represent standard deviations.

Shedding of FIPV DF-2 and PBFIPV-DF-2 was detected from day 3 p.i. to euthanasia of the PIP diseased animals at very low and variable amounts of an average value close to the detection limit of the genomic qRT-PCR (1.9×10^1^ FCoV RNA copies per µl fecal extract) (Fig. 1) with undetectable virus replication using the subgenomic qRT-PCR assay (data not shown). Virus shedding decreased to undetectable levels from day 21 p.i. in the PBFIPV inoculated animals.

The PBFIPV-DF-2-R3i infected cats began to shed the virus from day 3 p.i., virus shedding peaked at day 7 p.i. with 8.3×10^5^ FCoV RNA copies per µl fecal extract, remained high until day 14 p.i., then began to decrease until reaching 1.2×10^2^ FCoV RNA copies per µl fecal extract at day 35 p.i., and remained at this level until the end of the experiment (Fig. 1).

### FCoV Viremia

**Figure 2:**
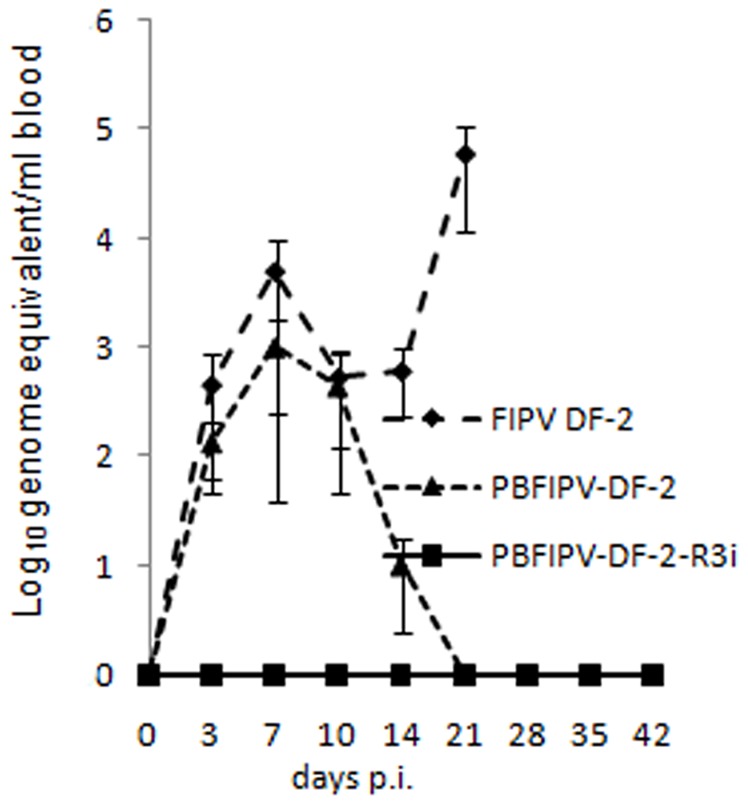
FCoV viraemia of cats challenged oronasally with the parent virus FIPV DF-2 (n = 4) and recombinant FCoVs PBFIPV-DF-2(n = 4) and PBFIPV-DF-2-R3i (n = 4), as monitored with genomic qRT-PCR. The means of groups are given. Error bars represent standard deviations.

A classic biphasic viremia was observed in FIPV DF-2 infected cats. FCoV RNA was detected from day 3 p.i., reached a first peak of 4.8×10^3^ FCoV RNA copies per ml blood by day 7 p.i., then decreased quickly after the emergence of neutralizing antibodies. A second wave of viremia was detected from day 14 p.i. until death peaking at 5.8×10^4^ FCoV RNA copies per ml blood (Fig. 2).

The PBFIPV-DF-2 infected cats developed only the first phase of viremia, FCoV RNA was detected in blood from day 3 p.i., reached a peak of 1×10^3^ FCoV RNA copies per ml blood by dy 7 p.i., and decreased to undetectable level at day 21 (Fig. 2).

In cats inoculated with PBFIPV-DF-2-R3i, complete absence of viremia was observed, the presence of FCoV genomic RNA in blood was not detected until the termination of the experiment (Fig. 2).

### FCoV Viral Load in Tissues

**Figure 3:**
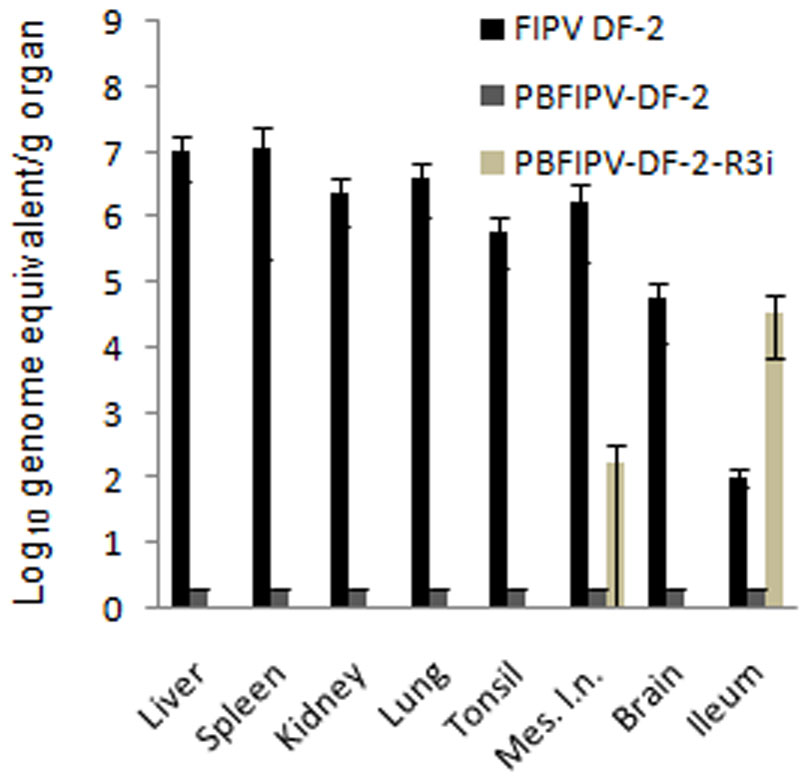
FCoV load of organs of cats challenged oronasally with the parent virus FIPV DF-2 (n = 4) and recombinant FCoVs PBFIPV-DF-2 (n = 4) and PBFIPV-DF-2-R3i (n = 4), as monitored with genomic qRT-PCR. The means of groups are given. Error bars represent standard deviations.

Cats infected with FIPV DF-2 showed high viral load (4.6×10^4^–1.2×10^7^ genomic RNA copies per g tissue) in the examined organs with intensive virus replication but very limited RNA copy numbers (1.1×10^2^ genomic RNA copies per g tissue) were obtained from the gut (Fig. 3). In cats infected with PBFIPV-DF-2, no detectable FCoV RNA copies were found in the examined organs.

The PBFIPV-DF-2-R3i challenged animals tested highly positive (3.6×10^4^ genomic RNA copies per g tissue) for FCoV RNA in the ileum. In addition, significantly lower level of positivity was observed in the mesenteric lymph node of two cats (3×10^2^ genomic RNA copies per g tissue) (Fig. 3). No other organs contained genomic RNA. The subgenomic qRT-PCR showed replication only in the gut.

High copy number (>10^3^) genomic qRT-PCR results were confirmed with subgenomic qRT-PCR assay not only from organs but all fecal and blood samples (data not shown).

### Humoral Immune Response

**Figure 4:**
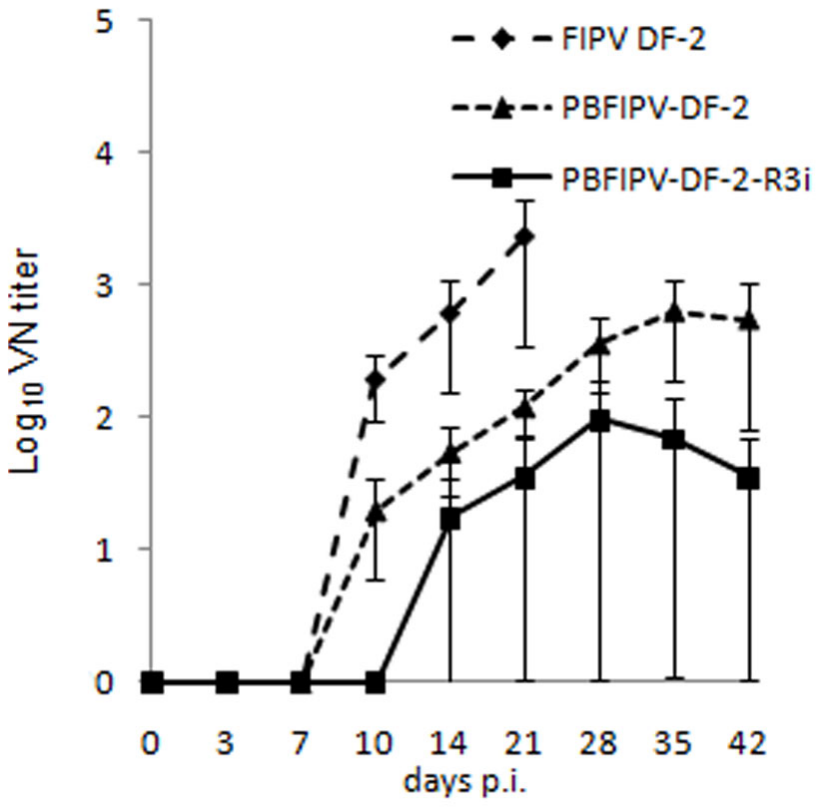
Induction of FCoV-neutralizing antibodies after oronasal challenge of cats with the parent virus FIPV DF-2 (n = 4) and recombinant FCoVs PBFIPV-DF-2(n = 4) and PBFIPV-DF-2-R3i (n = 4). The means of groups are given. Error bars represent standard deviations.

According to the pre-experimental data, no FCoV antibodies were detected at day 0 p.i. in any cat sera using ELISA and VN. In the FIPV DF-2 inoculated animals, neutralizing antibodies appeared by day 10 p.i., and reached high titers (2.4×10^3^) at the time of euthanasia (Fig. 4).

The PBFIPV-DF-2 challenged cats developed neutralizing antibodies from day 10 p.i. that elevated to lower levels (6.4×10^2^) by day 35 p.i. than in the FIPV DF-2 inoculated animals (Fig. 4). The difference between VN titers generated after FIPV DF-2 and PBFIPV-DF-2 was statistically significant (p = 0.037).

The PBFIPV-DF-2-R3i infected cats showed variable results. As ELISA and VN assays showed, two animals did not seroconvert (data not shown). Two animals seroconverted by day 14 p.i., and their VN titers remained at low levels (9.6×10^1^) compared with those of the PBFIPV-DF-2 infected cats (Fig. 4). The difference between VN titers generated after PBFIPV-DF-2 and PBFIPV-DF-2-R3i was statistically significant (p = 0.013).

## Discussion

The distinctive factor of the different pathogenesis of the two FCoV biotypes is the increased macrophage tropism of FIPV [10], [21], while FECV is tropic for the mature intestinal epithelium [22], [23]. Although alterations of several different genes of FCoV are suspected in the background of phenotypic characteristics, the most widely accepted theory suggests the possible role of the truncated ORF3abc in the altered tropism and consequent pathogenesis of the two biotypes [9], [14], [16], [18], [24]. However, an identical FCoV pair differing only in the intactness of ORF3abc has not been tested yet *in vivo* due to the lack of a cell culture for propagation of type I FECV and a “true” type II FECV isolate [8]. Our previous *in vitro* experiments added further evidence to the involvement of truncated ORF3abc to the increased macrophage tropism of type II FCoV [17]. In the present study, characterizing the parent FIPV DF-2 and the recombinant FCoV pair in *in vivo* experiments, we were able to distinguish significant differences in their biological properties.

Development of typical clinical signs and post mortem lesions of classical FIP were observed in cats infected with the parent virus FIPV DF-2 similarly as it was reported earlier [5], [18], [25]. Unexpectedly, kittens inoculated with PBFIPV-DF-2 showed only the acute phase of the disease with similar tropism as its wild-type parent FIPV DF-2. Sequencing of the pBFIPV-DF-2 infectious clone [17] and the recovered virus PBFIPV-DF-2 that was passaged in FCWF three times revealed point mutations originated in two waves during cloning (24429 (G/A) and 26064 (T/C)) and passaging (3098 (A/G), 5241 (G/A),7632 (C/T), 27817 (C/G) and 28492 (G/C)).

One of the two aa substitutions affecting nsp3 was found in papain-like protease 2 responsible for proteolytic processing of nsp1 and nsp2 [26], proteins with functions of host gene expression inhibition [27] and cellular signaling disruption [28].

One hydrophobic amino acid change (V to I) was identified close to the second heptad repeat in the interhelical region of S2 fusion domain of S protein responsible for virus-mediated membrane fusion. Similar conservative (M to L) aa substitution is suspected to be responsible for attenuation of of type I FCoV close to the first heptad repeat in this protein [11].

A nucleotide substitution found in the genome of PBFIPV-DF-2 affected the TRS of ORF7 gene. Mutations in TRS of transmissible gastroenteritis virus (TGEV) were shown not to alter virus replication *in vitro* [29]. Accordingly, in our *in vitro* experiments, no difference was found between the replication dynamics of the parent strain and the recombinant virus [17], and ORF7 mRNA transcription was found equal after both FIPV DF-2 and PBFIPV-DF-2 infection.

The 7b protein is considered as one of the virulence factors of FIPV [13], [18], [30], [31]. However, several FIPVs have lost virulence upon tissue culture passage do not have 7b mutations. A large number of FIPV and FECV genomes in GenBank also confirm that mutations in 7b are not associated with the FIP mutation. Considering that the exact biological function of this protein is unknown, and no single aa mutation in 7b was reported which alter virulence of FCoV, the role of the aa substitution (K/N) found in the 7b protein of PBFIPV-DF-2 is most likely has no effect on the virulence of this cloned virus.

The difference in virulence could be the consequence of any or combination of the point mutations in the genome of the recombinant FCoV obtained from the infectious clone [17] and during the three passages on FCWF after transfection and virus recovery. Similar problems with the attenuation of a virulent type I FCoV after bacterial cloning have been also reported by others [32]. A transient fever is often seen during the first few days after infection with virulent FIPVs, probably due to early host/virus interactions, but actual disease signs of FIP only occur when antibodies start to appear. The aforementioned mutations could lead to less effective replication of PBFIPV-DF-2 in macrophages/monocytes *in vivo*, or this mutated FCoV was not reacting in the same manner with antibodies as its wild type counterpart.

The low and inconsistent level of fecal shedding following inoculation with the parent FIPV DF-2 strain and the recombinant PFIPV-DF-2 containing truncated ORF3 is similar to that of previous observations [14], [24]. A classic biphasic viremia detected in FIPV DF-2 infected cats was similar to earlier experiments using the genetically closest FIPV strain 79–1146 [5]. As it was suspected from clinical signs, viremia was different in PBFIPV-DF-2 challenged animals. The infection kinetics of the two viruses was similar in the first days of infection, with the first replication peak at day 7 p.i. (Fig. 2), confirming our previous *in vitro* data obtained from feline blood monocytes [17], although the titer of PBFIPV-DF-2 was almost one log lower at the day of peak, and decreased rapidly. Seroconversion also started at the same time in the FIPV DF-2 and PBFIPV-DF-2 inoculated animals but remained at lower level in the latter case until the end of the experiment. These data indicate self-limiting replication of PBFIPV-DF-2 and complete clearance by the immune system that was further confirmed by the absence of the genomic RNA in the organs (Fig. 3).

The differences between the biological properties of the two viruses with truncated ORF3abc are substantial but by far less pronounced than it can be observed between the ORF3abc deleted and ORF3abc completed FCoVs. PBFIPV-DF-2-R3i genome was invariably absent in the blood monocytes. As a possible consequence of the absence of viremia, viral load of organs was not detected, the presence of PBFIPV-DF-2-R3i was found only in the mesenteric lymph node of two animals that showed weak seroconversion. These data indicate rather carrier role of macrophages/monocytes of FCoV with completed ORF3abc from the sites of the intensive FCoV replication. The absence of replication in blood monocytes of PBFIPV-DF-2-R3i inoculated cats coincide with previous data collected after FECV infection studies [3], [22], [23], [33], which clearly demonstrated the limited replication of the FECVs in mononuclear cells [5], [34].

The weak or missing seroconversion of PBFIPV-DF-2-R3i challenged cats is an obvious explanation of the low or absent systemic replication of the virus, and it is comparable with previous experimental type I FECV infections that showed low and often variable antibody titers in serum or plasma [22], [23], [35], [36], while a proportion of cats remained seronegative despite intensive fecal virus shedding [22].

Indeed, intensive fecal shedding and virus replication was detected during the whole period of the experiment and from the ileum of the sacrificed cats challenged with PBFIPV-DF-2-R3i carrying completed ORF3abc, in contrast to the other two investigated viruses. Our results are in accordance with the classic theory that mutations (deletions and nonsense mutations) altering the number and size of proteins translated from ORF3abc contribute to the altered tissue tropism of FIPV. This theory was reinforced by genetic investigations [24] that revealed that non-deleted ORF3abc of FIPVs accumulates four times more unique non-synonymous amino acid mutations in the ORF3abc than the FECVs, possibly modifying the biological function of these proteins.

Data of PBFIPV-DF-2-R3i shedding in the present study are similar with those of acute FECV and enteric CCoV infection. Recent experiments [32] with recombinant type II FIPV showed that ORF3c containing stop codon can be restored to code full-length 3c protein by point mutation during replication in internal organs and the gut. To investigate the mutability of this region we sequenced the 3c region of PBFIPV-DF-2-R3i from fecal and intestinal samples and we found no genetic alterations in this region (data not shown).

In type I FECV challenge studies [23], [35], [37], [38] FCoVs with intact ORF3abc shed at significantly higher titers compared with FIPVs, allowing horizontal spread to contact animals. The type II FCoVs used in our challenge studies are the result of double recombination between type I FCoV and type II CCoV [39]. In this respect, results of the present study are also in accordance with CCoV shedding pattern gained from canine experimental infection [40].

Summarizing the results of our challenge experiment, and comparing with previous experimental and clinical data, we conclude that the ORF3abc truncated recombinant virus showed attenuated phenotype with low virulence, transient viremia and complete clearance of the virus. Therefore, we cannot draw clear conclusion on the role of truncation of ORF3abc in the development of FIPV pathogenesis, although the biological properties of PBFIPV-DF-2 were closer to attenuated FIPV than to those of FECV. However, completion of ORF3abc vested PBFIPV-DF-2-R3i with biological properties that differentiate between the FECV and FIPV biotypes, such as intensive replication in the gut, absence of viremia and weak or no serological response.

The observation of numerous natural and experimental FCoV infection in the past decades led to the rise of the idea that completed ORF3abc is indispensable for intestinal replication of the virus. However, this theory has never been confirmed due to the lack of an identical virus pair differing only in their ORF3abc regions. Using such a virus pair our study is the first experimental proof which confirms the decisive role of ORF3abc in the intestinal replication of FCoV.
